# Selection for tameness alters play-like behaviour in red junglefowl in line with effects of domestication

**DOI:** 10.1098/rsbl.2024.0607

**Published:** 2025-02-05

**Authors:** Rebecca Oscarsson, Johanna Gjøen, Per Jensen

**Affiliations:** ^1^IFM Biology, AVIAN Behaviour Genomics and Physiology Group, Linköping University, Linköping 581 83, Sweden

**Keywords:** tameness, domestication, play behaviour

## Abstract

The phenotypic alterations brought by domestication have been hypothesized to be driven by selection for tameness. To explore this, we selected red junglefowl (RJF) for high (HF) and low (LF) fear of humans for 14 generations. We previously found that domesticated chickens performed more play-like behaviours during early ontogeny, and therefore, in this study, we explored potential effects of tameness. Groups of three to four chicks were randomly created from each selection line, and each group was moved to an enriched play arena twice per week, from day 6 until day 53 post-hatch. The frequency of 14 different play-like behaviours, categorized as locomotor, social and object play-like behaviour were recorded for 30 min at every observation instance. Every group of three or four birds constituted the independent statistical replicates and measures were averaged within the groups. The frequency of total play-like behaviour as well as object, and locomotor play-like behaviour was significantly higher in LF, while social play-like behaviour was significantly more common in HF. This largely mirrors previous observations of differences between domesticated and ancestral chickens. Hence, our results support the important role of tameness for the evolution of domesticated behaviour.

## 1. Introduction

Phenotypic alteration caused by domestication includes changes in behaviour, physiology and appearance [1,2], and is often referred to as the domestication syndrome. Behavioural changes that are part of this syndrome include increased prosociality [3] and playfulness [4]. Domestication is an example of a rapid evolutionary process and can therefore serve as a powerful model to understand details about correlated traits in evolution at large. Chickens were domesticated supposedly around 8−9000 years ago from the ancestral red junglefowl (RJF) [5,6], a social bird living in groups of multiple males and females [7], and their behaviour has been altered in line with the domestication syndrome [8], making them excellent study subjects for this type of research.

Belyaev [9] hypothesized that tameness is the main selected trait that drives the domesticated phenotype. The logic here is that tameness would make animals more tractable and possible to breed under the auspice of humans, and tameability would therefore be a necessary first step in the domestication process. In his studies, utilizing farm foxes, selection for tameness caused a cascade of phenotypic alterations within few generations that were in line with the domestication syndrome, for example, loss of pigmentation, floppy ears and modified ontogenetic development. However, a major shortcoming in the studies of Belyaev is the fact that foxes have not previously been domesticated, and therefore, there is no present-day domesticate to compare the effects with. Hence, it is difficult to conclusively state that tameness drives the domestication syndrome based on this experiment.

We have tackled this problem by studying the ancestor of domestic chickens, the RJF. The hypothesis that reduced fear of humans can generate many of the domestication-related changes seen in modern chickens can therefore be tested by selecting these wild birds for increased tameness and monitoring correlated phenotypic alterations. The logic behind the hypothesis is that tameness is presumably a necessity for a successful life among humans, and therefore, we have selected RJF for either high (HF) or low fear (LF) of humans in a standardized fear-of-human test for 14 generations. This selection for increased tameness has caused correlated changes in several traits associated with the domesticated phenotype, such as modified sociality, increased hatch weight and growth and decreased brain size [8,10,11].

One important behaviour known to have been affected by domestication in several species is play (e.g. [12]), and recently, we found similar results in chickens, where domestic birds perform play-like behaviours significantly more than ancestral RJF [13]. Overall, the common similarity across species is that domesticates play more than their ancestors; however, the results vary between different play categories. Play behaviours are commonly categorized as locomotor play (e.g. frolicking and running), social play (e.g. rough-and-tumble play, sparring) and object play (involving interaction and manipulation of different items; [14]). Interestingly, domestication increased social play in rats, while the opposite was found in chickens. Nonetheless, we hypothesized that this overall increase in play-like behaviour, like other aspects of the domestication syndrome, may also be driven by increased tameness.

Play is widespread among mammals and birds [15]. Regarding avian species, play is most prevalent in altricial birds, such as parrots and corvids [16–18], but has also been suggested to be present in precocial birds, such as chickens [19–21]. However, there is a lack of consensus on how to unanimously identify play in various species. In the absence of a widely accepted and reliable definition, it has been suggested that behaviours should meet five criteria to be identified as play [22] (i) it lacks immediate function and purpose to the animal; (ii) it brings reward or pleasure to the performer; (iii) it differs from functional expression of behaviour in form or temporal organization; (iv) it is performed repeatedly, in a non-stereotypic manner, throughout at least parts of the ontogeny; and (v) it is primarily initiated when the animal is in a healthy and stress-free state. Whether chickens show behaviour that fully meets these criteria can still be questioned, but some types of juvenile activities certainly appear to do so, as shown in our earlier studies [13].

We previously adopted the aforementioned categories when studying domestication effects on the ontogeny of play-like behaviours in young chickens. In that study, RJF chicks engaged more in locomotor and social play-like behaviour, while the domesticated white Leghorn (WL) chicks engaged more in object-directed behaviour, and overall showed significantly more play-like behaviour [13]. The aim of the present study was to explore whether selection for tameness alone affects play-like behaviours in line with that caused by domestication. We compared RJF selected for LF and HF of humans and predicted to find differences similar to the ones found between domesticated and ancestral birds. More specifically, we expected that LF birds would perform more object-directed play-like behaviour and show more play-like behaviour overall, while HF birds would perform more social and locomotory play-like activities.

## 2. Material and methods

### (a) Animals

We used RJF from generations 13 and 14 from lines selected for LF or HF. Both lines had their origins in an outbred group of two different zoo populations and had in every generation been selected based on a fear score derived from a fear-of-human test (detailed information about the breeding and selection programme is provided in [23]). Briefly, the birds were tested individually in a standardized fear of human test at 12 weeks of age, where their behavioural responses (e.g. vigilance, vocalizations, flight attempts) to a moving human were recorded in a 1 × 3 m large arena. The most and least fearful birds were selected for breeding in each generation. The birds were 7.5 weeks old by the end of this study and had therefore not yet been tested for fear of humans.

The birds were bred in three batches (LF; batch 1: *n* = 7, six males and one female, batch 2: *n* = 11, seven males and four females, batch 3: *n* = 20, 12 males and eight females. HF; batch 1: *n* = 28, eight males and 20 females, batch 2: *n* = 27, 15 males and 12 females, batch 3: *n* = 20, 10 males and 10 females), all incubated and housed under the same conditions in the facilities at Linköping University. The eggs were all incubated and hatched in darkness, in the same small incubator set to 37.8°C, 55% relative humidity and with hourly rotation. Three days before hatch, the eggs were moved to hatching trays with hatcher settings of 37.5°C and 65% relative humidity.

### (b) Housing

The chicks were housed in sex-mixed groups of 7−12 individuals each (in total 2−3 play-groups each in one pen; see below), with HF and LF kept in separate pens. The pens consisted of solid floor cages (W × L × H: 0.7 × 0.68 × 0.57 m) and were provided with sawdust, a heat roof, as well as feed and water ad libitum. At four weeks of age, the heat roofs were removed and replaced with perches. Since young chicks do not form strict hierarchies, we did not record individual data but treated each play-group as a statistical unit (see below).

### (c) Experimental set-up and procedure

After being wing tagged and vaccinated (day 1), random, sex-mixed play-groups of three or four birds were created within each cage, using coloured leg rings of unique codes. Each such play-group constituted the test unit and was always tested together. The chicks were tested at the following days of age: 6, 8, 10, 15, 18, 22, 25, 29, 32, 36, 39, 43, 46, 50 and 53.

To enable a proper comparison, the idea was to replicate our previous experiment comparing RJF and domesticated chickens [13]. Therefore, the tests occurred under identical conditions as in that experiment. For each test session, an entire play-group was moved from its home pens and placed in a play arena with the lights off (complete darkness). The completely enclosed soundproof play arenas were considerably larger than the home pens (L × W × H: 1.17 × 0.8 × 1 m), containing sawdust, a small pile of hay, a perch along one short end and a chain hanging from the ceiling. Four identical arenas were situated in the laboratory, allowing four play-groups to be tested at the same time. Once one group had been placed in each arena, the test session was started by the light being turned on simultaneously in all arenas.

Each test session lasted 30 min and was recorded through overhead video cameras. After 10 min, a fake rubber worm was presented to the birds to provide further play objects. The first fake worm used (2 × 60 mm) was replaced with a larger one (3 × 165 mm) on test day 5 (when the chicks were 18 days old). When 20 min had passed, a small cardboard box with three live mealworms was inserted in the arenas. These objects were inserted through a small hatch in the corner of the arena, so the chicks were not able to see the experimenter.

The same ethogram as in our previous study [13] was used, and the behaviours were grouped the same way. From the videos, the occurrence of 14 play-like behaviours was scored and thereafter grouped into three categories: locomotor, social and object play-like behaviour. Moreover, all occurrences of all behaviours were summed into the category of total play-like behaviour. The behaviours were summed in the same way as in the previous study, i.e. locomotor play-like behaviour included running, frolicking, wing flapping, spinning and spinning while wing flapping. Object play-like behaviour included object running, worm running, object/worm chasing, object/worm exchange and worm pecking. Social play-like behaviour included sparring jumping with or without contact, and sparring stand-off with or without contact.

A complete ethogram with detailed descriptions of all included behaviours is provided in electronic supplementary material, table S1. Line graphs of each behaviour are provided in electronic supplementary material, figure S2. Video footage showing examples of the different play-like behaviours is provided in the supplementary material of [13].

## 3. Sampling and data analysis

The behaviours were recorded in the same way as in our previous study [13]. All videos were scored by the same observer (the first author). The behaviour was recorded in 15 s segments throughout the 30 min, using 1/0 sampling for each chick. Specifically, for every time segment of 15 s, it was recorded how many of the three or four individuals that engaged in each behaviour at least once. As a result of this, in every segment, each behaviour got a score from 0 to 3 or 4 (depending on the size of the play-group), and for the entire 30 min test, the total value of each behaviour could vary from 0 (if the behaviour was never observed) to 360 or 480 (meaning that all three or four birds performed the behaviour once or more during all 15 s segments). This was then transformed into a percentage to make all recordings comparable regardless of group size.

Each group of three or four birds constituted an independent statistical replicate. Generalized linear mixed models with a repeated measures design were used to analyse the effects of age, line and their interactions. Age was taken into the model as a categorical factor, not a linear covariate, since the expected ontogenetic pattern was an inverse U-shape. The sex ratio in each group, measured as the percentage of males, was also included as a fixed effect, owing to the unintentional uneven distribution of males and females in the two treatments. The model was fitted for normal probability distribution with identity link function. The data are presented as mean percentage of observations per group with standard error of the mean. The statistical analyses were performed in SPSS 29.0.0.0.

## 4. Results

**Figure 1.**
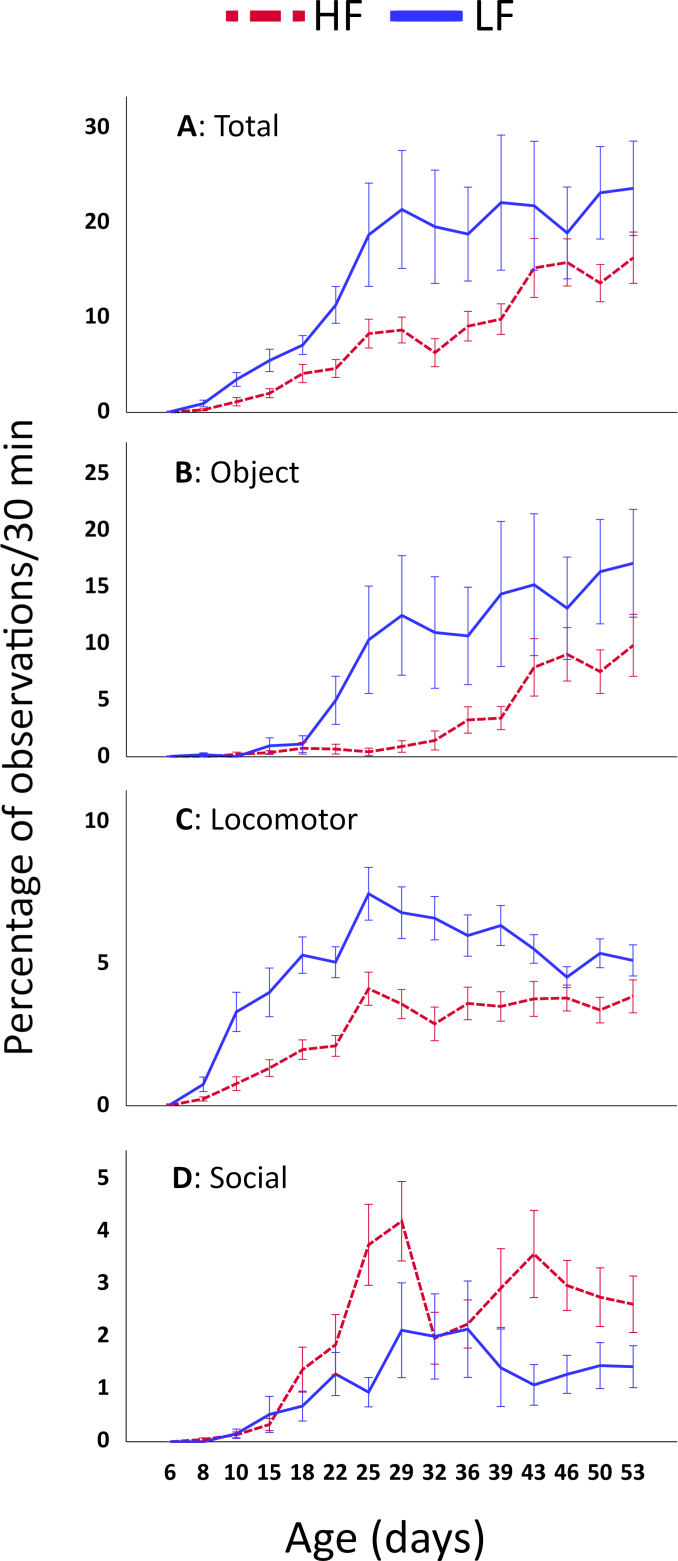
Mean per cent of observations (± s.e.) per group per 30 min of (A) total play-like behaviours, (B) object-directed play-like behaviour, (C) locomotory play-like behaviour and (D) social play-like behaviour, at different ages, in RJF chicks selected for high (HF) and low (LF) fear of humans. Note that the scales differ between the graphs.

All 14 behaviours were recorded in both lines. The frequencies of each of them over time are shown in electronic supplementary material S2. The frequency of total play-like behaviour (sum of all behaviours) showed a significant change with age in both lines (figure 1A and table 1). LF performed significantly more total play-like behaviour compared with HF, and there was a significant interaction between line and age (figure 1A and table 1), caused by a difference between the lines in age-related developmental pattern.

**Table 1.** Results of fixed effects test performed on generalized linear mixed models for total, object, locomotor and social play.

play-like category	model term	*F*_1, 14_	*p*‐value
total	line	43.979	<0.001
	age	43.426	<0.001
	sex ratio	0.253	0.615
	line × age	5.437	<0.001
object	line	38.181	<0.001
	age	13.227	<0.001
	sex ratio	0.229	0.633
	line × age	3.750	<0.001
locomotor	line	123.876	<0.001
	age	98.111	<0.001
	sex ratio	2.852	0.092
	line × age	10.371	<0.001
social	line	19.870	<0.001
	age	17.524	<0.001
	sex ratio	0.000	0.997
	line × age	2.078	0.012

Object-directed behaviour was the most common play-like behaviour category (figure 1B), followed by locomotory (figure 1C) and social play-like behaviour (figure 1D). Object and locomotor behaviour was more frequent in LF (figure 1B*,*C and table 1), and social play-like behaviour was more frequently performed by HF (figure 1D and table 1). All three subtypes showed significant age effects (table 1). Moreover, there were significant interactions between line and age in all categories (table 1), confirming the difference in age-related development. As can be seen in electronic supplementary material, figure S2, the pattern was similar for each of the 14 different play-like behaviours, i.e. the ones categorized as object directed and locomotory were more frequent among the LF, whereas those categorized as social were more frequent among the HF. The sex ratio in the groups had no effect on any of the categories (table 1). The same was the case for each of the separate behaviours, except ‘frolicking’, which was performed less in groups with more females than males (electronic supplementary material, figure S2).

## 5. Discussion

To our knowledge, the present study is the first in any species that has investigated how play-like behaviour is affected by selection for increased tameness. The results show that such selection has indeed affected such behaviour and some of the differences, but not all, between the selection lines are similar to what we have previously found when comparing domesticated chickens with their ancestors [13]. This supports the hypothesis that tameness is an important driver of the domesticated phenotype. Similar to the previously described effects of domestication, object-directed play-like behaviour was the dominant category causing this difference. Social play-like behaviour also followed a similar pattern, with the HF birds performing this more, mirroring previously demonstrated differences between ancestral and domesticated chickens. However, contrary to our expectations, locomotory play-like behaviour was more frequent in the LF, in contrast to previous observations that RJF show more of this than domesticated chickens.

Domestication has caused a range of phenotypic effects, many of which tend to have evolved in most domesticated species. This is referred to as the domestication syndrome, consisting of, for example, loss of pigmentation, changes in body size, morphological alterations (e.g. brachycephaly and chondrodystrophy), reduced brain size and various changes in behaviour [2]. Belyaev [9] launched the hypothesis that this complex of similar phenotypic alterations may have been driven by the reduction in fear of humans that must logically have been a necessity during early domestication and showed that selecting silver foxes for increased tameness indeed caused a cascade of domesticated phenotypes in the tamer selection lines. Similarly, we have found that selecting ancestral RJF for reduced fear of humans has caused correlated changes in, for example, body size, brain mass and social behaviour [8,10,11], corroborating Belyaev’s hypothesis. The main advantage of using chickens rather than foxes as a model is that we can compare the results of the selection for tameness with the modern domesticates and thereby make a more informed test of the hypothesis, and indeed most of the correlated effects we have previously reported mirror the differences between present-day domesticated chickens and ancestral RJF. Here, we focused on a relatively overlooked aspect of the domestication syndrome, i.e. play-like behaviour.

Play has been reported in juveniles, and some cases in mature subjects, in many vertebrate species, and even in some invertebrates [15,24]. Moreover, in some species, play has been shown to have important functions both during immature stages of development and among adults [25,26]. The frequency of play has been shown to be higher in domesticated strains compared with their wild ancestors, for example in rats [27], guinea pigs [28], dogs [29] and chickens [13]. However, this does not necessarily affect all aspects of play similarly, since social play is more common in domesticated than in wild rats [27], whereas ancestral RJF on the contrary perform more play-like social behaviour than domesticates [13]. The reason for such differences between species is not known but could possibly reflect the fact that play serves different functions related to the social structures of different species. For example, in chickens, we have previously found that young chicks that had been stimulated to perform more social play-like behaviour were more successful in a social competition situation later in life [30]. Although speculative, it is possible that social play may serve an important function for wild RJF to develop their competitive abilities, which perhaps is not as crucial for wild rats. There is an urge for more research on the functions of play in chickens, since some of our results are not in line with what has been found in some mammals (e.g. [12]). For example, studies should focus on how different categories of play affect later cognitive abilities in different contexts, such as social interactions, spatial orientation, etc.

In the present study, we found that selecting RJF for reduced fear of humans for 14 generations caused a similar modification to total play-like behaviour as seen when comparing domesticated chickens to their ancestors, i.e. tamer birds showed significantly more such behaviour than non-tame birds when all different activities were lumped together. With respect to the different categories, object-directed behaviour was more common in the tamer birds, and social play-like behaviour was more common in the non-tame birds, mirroring the fact that the same categories are more common as when domesticates are compared with RJF [13]. However, the tamer birds showed more locomotory play-like activities, which is opposite to the fact that RJF have more of this activity than domesticates [13]. Assuming that the play-like behaviours we have recorded actually represent play in the same sense as corresponding behaviour in young mammals, the results indicate that play may not be a uniform behaviour system, but that different types of play may serve different functions and respond independently to genetic selection. Regardless, this is the first study showing an effect of increased tameness on play-like behaviour in any species, and strongly supports the idea that reduced fear of humans is a driver of many aspects of the domestication syndrome.

The functions of play are not yet fully understood. It is a costly behaviour that does take up a large portion of the time budget of many young vertebrates and should therefore also be associated with clear benefits outweighing the costs. The most common theories suggest that play is important for developing motor skills and cognitive abilities [15,31]. Few empirical studies have been performed to test these theories, but we recently reported that stimulation of social play in chickens improved their later social competitiveness [30]. More research is clearly needed to reveal the functions of play in general, and the reason for the overall increase during domestication and as a result of increased tameness.

Play mainly occurs when stress levels are low or absent [22]. Therefore, one possible factor that could be related to the correlated responses in play-like behaviour is the decreased fear and stress responses in domesticated birds as well as in the RJF selected for increased tameness [32]. In domesticated WL compared with RJF, this has been linked to changes in the expression of certain genes in the hypothalamus–pituitary–adrenal (HPA) axis, suggesting a higher capacity of negative feedback within the HPA-axis as well as reduced synthesis of corticosterone in the adrenal glands [33]. We have also previously shown that the HPA-axis tends to be less reactive in the tamer RJF from the selection lines studied here [34], corroborating the hypothesis that lower stress levels could be a factor explaining the effects observed here.

The correlated responses may also be related to changes in brain size. Brain size has been observed to correlate with the increased occurrence and complexity of play in some native Australian birds [35]. Domesticated chickens have a smaller relative brain mass; however, a larger relative mass of cerebellum compared with RJF [36], and in analogy, RJF selected for reduced fear of humans have a smaller relative brain mass but a larger relative cerebellum mass compared with less fearful birds [37]. The cerebellum is associated with motor control [38] and social processing [39], both of which may, in turn, be related to play behaviour.

Sex differences in chicken play ontogeny have been identified [40], and males tend to play more than females. Unintentionally, in HF we had more females than males, and in the LF, there were more males than females. To address this, as the behaviour was not scored on an individual level, the sex ratio of each play-group was included as a covariate in the statistical model. No effect of sex ratio was found for the play categories, whereby any potential such influence can be dismissed. Unexpectedly, an effect of sex ratio was found for ‘frolicking’. This contradicts our previous finding, that no sex difference is present for locomotor play [40], hence the reason for this remains to be explored.

In conclusion, we found that RJF selected for reduced fear of humans play significantly more than more fearful birds. This mirrors previous findings that domesticated chickens play more than ancestral RJF and corroborates earlier studies showing that a wide range of domesticated phenotypes can evolve as correlated effects to reduced fear of humans. Not all play-like behaviour was affected as could be expected from what we know about differences between RJF and present-day domesticates, but adding our present results to previous findings emphasizes the importance of tameness as a driver for the domestication syndrome.

## Data Availability

The complete data set for the present study is provided in electronic supplementary material, table S3. The data set contains all raw data for the behavioural recordings of each of the groups during all sampling weeks. Supplementary material is available online [41].
